# Supplementary material for: The interactome of intact mitochondria by cross-linking mass spectrometry provides evidence for coexisting respiratory supercomplexes
Source: Mol Cell Proteomics. 2017 Dec 8;17(2):216–32. doi: 10.1074/mcp.RA117.000470 (PMC5795388; doi:10.1074/mcp.RA117.000470)
Supplement: Supplemental Data [file supp_RA117.000470_133922_0_supp_23978_fzffwf.zip › spectra_annotation/mito_DR_spectra_annotation/47-1-3-1-4-1.pdf]

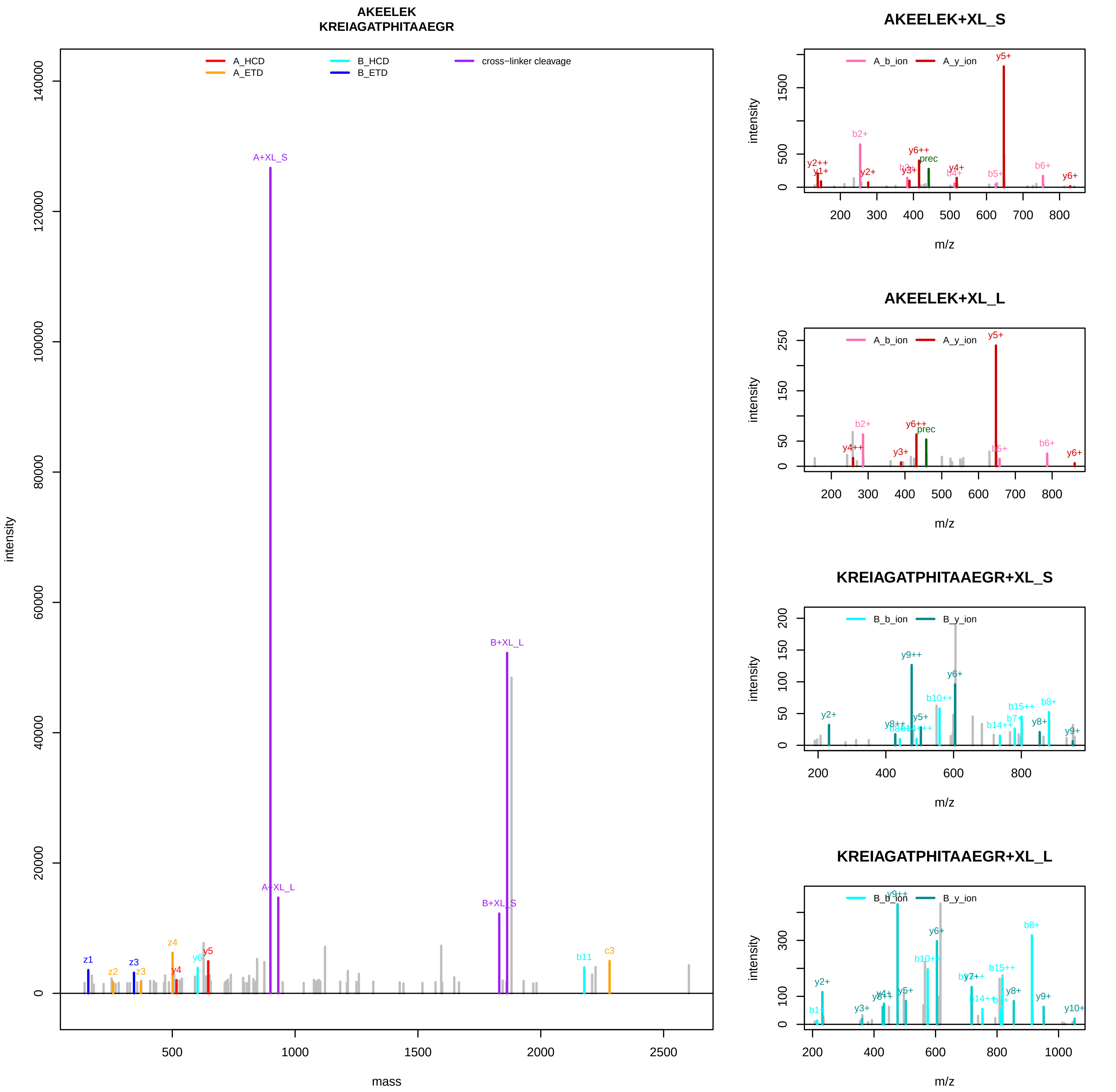

AKEELEK
KREIAGATPHITAAEGR
A_HCD
A_ETD
B_HCD
B_ETD
cross−linker cleavage
A+XL_S
A+XL_L
B+XL_L
B+XL_S
z1
z2
z3
z4
y4
y5
y6
b11
c3
intensity
mass
0
20000
40000
60000
80000
100000
120000
140000
500
1000
1500
2000
2500
AKEELEK+XL_S
AKEELEK+XL_L
KREIAGATPHITAAEGR+XL_S
KREIAGATPHITAAEGR+XL_L
A_b_ion
A_y_ion
B_b_ion
B_y_ion
prec
y5+
b2+
y6++
y2++
y1+
y2+
y3+
y4+
b4+
b5+
b6+
y6+
y4++
y9++
y6+
b10++
b15++
b8+
y2+
y8+
y9+
y5+
b14++
y3+
y10+
m/z
